# The relationship between structural characteristics and gambling behaviour: An online gambling player tracking study

**DOI:** 10.1007/s10899-022-10115-9

**Published:** 2022-05-13

**Authors:** Michael Auer, Mark D. Griffiths

**Affiliations:** 1 neccton GmbH, Davidgasse 5, 7052 Muellendorf, Austria; 2 International Gaming Research Unit, Psychology Department, Nottingham Trent University, 50 Shakespeare Street, NG1 4FQ Nottingham, UK

**Keywords:** Online gambling, Internet gambling, Structural characteristics, Event frequency, Behavioral tracking

## Abstract

Structural characteristics of games have been regarded as important aspects in the possible development of problematic gambling. The most important factors along with individual susceptibility and risk factors of the individual gambler are the structural characteristics such as the speed and frequency of the game (and more specifically event frequency, bet frequency, event duration, and payout interval). To date, the association between structural characteristics and behavior has not been studied in an online gambling environment. The present study investigated the association between structural characteristics and online gambling behavior in an ecologically valid setting using data from actual gamblers. The authors were given access to data from a large European online gambling operator with players from Germany, Austria, UK, Poland, and Slovenia. The sample comprised 763,490 sessions between November 27, 2020 and April 15, 2021 utilizing data from 43,731 players. A machine learning tree-based algorithm with structural characteristics and session metrics explained 26% of the variance of the number of games played in a session. The results also showed that only 7.7% of the variance in the number of bets placed in a session was explained by the game’s structural characteristics alone. The most important structural characteristic with respect to the number of games played in a session was the event frequency of the game followed by the maximum amount won on a single bet in a session.

## Introduction

In addition to biological and psychological factors, structural characteristics of games have been regarded as important aspects in the possible development of problematic gambling (Griffiths, 1993). Structural characteristics refer to the inherent features of games (e.g., jackpot size, stake size, event frequency) as well as mathematical aspects of a game such as the probability of winning and return to player (RTP) (Goodie, 2015; Griffiths, 1993; Griffiths & Auer, 2013; Lopez-Gonzalez et al., 2019; McCormack & Griffiths, 2013). RTP is the percentage of the amount wagered that is paid back as winnings. For example, ‘classic’ roulette with 36 numbers and a zero has a RTP of 97.3%, given an infinite number of games. This percentage might of course be higher or lower for a smaller number of observations and might never reach the actual figure of 97.3% for a single player.

Auer and Griffiths (2013) have argued that the acquisition, development, and maintenance of problem gambling is independent of the type of game (e.g., slot machines, scratchcards, roulette, etc.). They argued that the most important factors along with individual susceptibility and risk factors of the individual gambler are the structural characteristics such as the speed and frequency of the game (and more specifically event frequency, bet frequency, event duration, and payout interval).

Apart from the mathematical aspects, such as reinforcement schedules and betting configurations, structural characteristics such as the theme of the game, pseudo-skill buttons, color, lights, and sound effects may also have an impact on player behavior (Goodie, 2015; Griffiths, 1993; Lopez-Gonzalez et al., 2019; McCormack & Griffiths, 2013; Parke & Griffiths 2006, 2007). The preference for games has shown to be associated with individual factors (e.g., traits and motives), as well as situational factors (e.g., availability, accessibility and diversity games offered) (Smith et al. 2007).

Theoretical and empirical research supports the notion that structural characteristics influence gambling behavior. One crucial aspect of gambling is the chance to win, which is directly linked to game features such as the chance of winning and hitting the jackpot (Binde 2013). Operant and classical conditioning to specific structural characteristics might also play an important role regarding the intensity of gambling behavior (Blaszczynski & Nower, 2002).

The relationship between the reward characteristics of games of chance and gambling behaviors has been studied both by laboratory studies and self-report studies (e.g., Coates & Blaszczynski 2013; Dixon et al. 2006; Haw 2008; Livingstone & Woolley 2008; McCormack & Griffiths 2013; Weatherly & Brandt 2004). In a telephone survey of Australian gamblers, Livingstone and Woolley (2008) found that reward characteristics (size and frequency of wins) and free games (such as free spins) were the most attractive structural characteristics of a game. However, Weatherly and Brandt (2004) failed to find an association between gambling behavior and reward characteristics. Other studies (e.g., Coates & Blaszczynski, 2013; Dixon et al., 2006) have reported a significant relationship between gambling behavior and reward characteristics. Dixon et al. (2006) found that gamblers prefer games with more frequent but smaller wins to games with less frequent and big wins and is in line with the findings of Coates and Blaszczynski (2013).

Delfabbro and Winefield (1999) studied in-session gambling in an observational study of 21 occasional poker-machine players. Larger wins were found to disrupt response rates giving rise to larger post-reinforcement pauses, whereas response rates were maintained by small rewards. Sharpe et al. (2005) designed eight experimental gambling machines and studied 210 participants. They assessed problem gambling (with the South Oaks Gambling Screen [SOGS]). More problem gamblers than non-problem gamblers used high denomination bill acceptors and bet over one-dollar per wager. They concluded that the reduction of maximum bet levels was the only modification likely to be effective as a harm minimization strategy for problem gamblers. Livingstone and Woolley (2008) found that gamblers chose bet size and number of lines in slot machines to maximize their frequency of wins.

As far as the present authors are aware, only one study which has examined the relationship between structural characteristics and gambling behavior in a real-world setting. Leino et al. (2015) had access to data from 31,109 Norwegian players who played on VLTs in January 2010. They defined the individual number of bets made across games as the dependent variable, whereas reward characteristics of a game (i.e., payback percentage, hit frequency, size of winnings, and size of jackpot) and bet characteristics of a game (i.e., range of betting options and availability of advanced betting options) were the independent variables. Their results showed that the number of bets was positively associated with payback percentage, hit frequency, being female, and age, and negatively associated with size of wins and range of available betting options. The reward characteristics and betting options explained 27% and 15% of the variance in the number of bets made, respectively.

To date, the association between structural characteristics and behavior has not been studied in an online gambling environment. Compared to land-based gambling, online gambling transactions are always assigned to one account and therefore (it is assumed) one individual. In recent years, behavioral tracking studies have investigated a number of different aspects of gambling and problem gambling. Ukhov et al. (2021) studied the difference between online casino and sports bettors (N = 10,000). They found that the number of cash wagers per active day contributed the most to problem gambling-related self-exclusion among online sports bettors, whereas the volume of money spent contributed most to problem gambling-related self-exclusion among online casino players.

Philander (2014) used tracking data (from a publicly available dataset used by Braverman and Shaffer [2012]) comprising a sample of online live action sports betting wagers to identify high-risk online gamblers and found that bet intensity (the average number of bets per day), variability (standard deviation of wagers), frequency (the number of betting days during the period of observation), and trajectory (a linear regression model with wager as a dependent variable and a sequence number as a predictor), as well as age and gender were insufficient variables to classify probable disordered gamblers with reasonable accuracy. Philander (2014) reported that a high classification accuracy was generally accompanied by a higher false positive rate.

Auer and Griffiths (2017) used player tracking data to compare self-reported gambling with actual gambling. They found that gamblers underestimated their losses and overestimated their winnings. Other studies utilizing player tracking data have researched the effects of voluntary limit setting (e.g., Auer & Griffiths, 2013), the effects of personalized feedback (e.g., Auer and Griffiths, 2015), and self-exclusion (e.g., Percy et al., 2016). More specifically, Auer and Griffiths (2013) found that in relation to controlling monetary spend, online casino players benefited more from voluntary money limits and online poker players benefited more from voluntary time limits. Auer and Griffiths (2015) found that online gamblers receiving personalized feedback spent significantly less time and money gambling compared to controls that did not receive personalized feedback. Percy et al. (2016) compared machine learning techniques for the prediction of self-exclusion of online gamblers and reported the random forest technique as being the most effective.

The present study investigated the association between structural characteristics and online gambling behavior in an ecologically valid setting using data from actual gamblers from five countries (Germany, Austria, UK, Poland, and Slovenia). The most recent surveys carried out in each of these countries shows that the prevalence of problem gambling is relatively low: 0.39% in Germany (Banz, 2019), 1.1% in Austria (Kalke et al., 2018), 0.6% in the UK (Gambling Commission, 2020), 1.1% in Poland (Lelonek-Kuleta et al., 2020), and 1.45% in Slovenia (Makarovič, 2010). Moreover, most data suggests that the prevalence of problematic online gambling is higher than that of offline gambling (Mora-Salgueiro et al., 2021) but it should also be noted that most online gamblers also gamble offline (Wardle et al., 2011).

The authors assumed that structural characteristics would be associated with gambling behavior. Based on previous research it was expected that gambling behavior would be positively associated with payback percentage, frequency of wins, size of wins, and the opportunity to vary the bet size of a bet in a game.

## Method

The authors had access to data from a large European online gambling operator with players from Germany, Austria, UK, Poland, and Slovenia. Each and every wager and every win at the respective point of time was available from November 27, 2020 until April 15, 2021.

### Procedure

Based on the raw transactions which comprised each wager and win, the authors computed the number of gambling sessions. If there was more than fifteen minutes between two wagers it was operationally defined as a new session for data analysis purposes. Wagers that occurred within 15 min or less of each other were counted as within session wagers. Only sessions during which one particular game type was played were used for further analysis. Sessions with more than one particular game type were discarded from the analysis. The authors only selected sessions with one game type, because the study was examining the effect of a particular game’s structural characteristics on specific outcome characteristics (i.e., the session’s theoretical loss and number of games played). If there was more than one game type played in a session it would not be possible to attribute the dependent variables to the structural characteristics of a specific game.

### Sample

The sample comprised 763,490 sessions between November 27, 2020 and April 15, 2021 utilizing data from 43,731 players. The average age was 43 years (SD = 13.11) and 17.4% were female. On average, men were aged 41.80 years (SD = 13.29) and women aged 43 years (SD = 12.18). In total, 598 different games were played. Only games with at least 100,000 wagers across the observational period were taken into account. In order to compute the mathematical metrics for each game, a specific number of observations needed to be available. The online gambling operator’s game portfolio consisted of six game groups (i.e., blackjack, live blackjack, roulette, live roulette, video poker, and slots games). The majority of the games played were slots games.

### Measures

A number of game characteristics were computed across all available transactions for each of the 598 games which had at least 100,000 wagers. These were:

- *Event frequency*: The event frequency is the number of seconds between two consecutive wagers. The smaller the event frequency the more frequent a game is being played.
- *Return to player (RTP*): The RTP is the percentage of the amount wagered paid back to players as winnings. The RTP is between 0 and 1. A higher value indicates that the game pays back out more to players.
- *Hit frequency*: The hit frequency is the ratio between the aggregate number of wagers made divided by the aggregate number of wins obtained by all players. A lower quotient represents more frequent wins per wagers in a game (i.e., fewer wagers needed to obtain a win of any size).
- *Continuity*: The continuity is the average session duration in minutes. The higher the continuity, the longer the players gamble on average on a particular game.
- *Average bet*: The average bet is the mean average amount wagered across all wagers for a particular game.
- *Standard deviation bet*: The standard deviation bet is the variation in amount of money bet across all wagers for a particular game.
- *Average win*: The average win is the mean average amount won across all winnings for a particular game.
- *Standard deviation win*: The standard deviation win is the variation in amount of money won across all winnings for a particular game.

For each session a number of metrics were computed. In contrast to the metrics above, the metrics below were calculated for each session across all the games played in that session (rather than for each game across all players). Some metrics (e.g., average bet, average win, RTP, etc.) can be computed for a specific game as well as for a session. The in-session metrics were:

- *Session length*: The total duration of the session in minutes.
- *Number of bets*: The total number of wagers in a session.
- *Amount bet*: The total amount of money wagered in a session.
- *Amount lost*: The amount lost was computed as the amount won minus the amount wagered. A negative value indicates that the player has lost in the session and a positive value indicates that the player won in the session.
- *Number of wins*: The number of times the player won in a session.
- *Amount won*: The amount of money a player won in a session.
- *Average bet*: The mean average bet across all wagers in a session.
- *Average win*: The mean average win across all wagers in a session.
- *Maximum bet*: The maximum amount of money bet across all wagers in a session.
- *Maximum win*: The maximum amount of money won across all wagers in a session.
- *Return to player (RTP)*: The RTP is the percentage of the amount wagered paid back to players as winnings. The RTP is between 0 and 1. A higher value indicates that the game pays back out more to players.
- *Hit frequency*: The hit frequency is the ratio between the aggregate number of wagers made divided by the aggregate number of wins obtained by all players. A lower quotient represents more frequent wins per wagers in a game (i.e., fewer wagers needed to obtain a win of any size).
- *Theoretical loss*: The theoretical loss is the amount of money the player would have lost based on the actual amount of money wagered and the game’s RTP (Auer, Schneeberger & Griffiths, 2012). The underlying computation for theoretical loss is the amount of money bet multiplied by 1 minus RTP.

### Statistical analysis

Linear regression analysis (Massaron & Boschetti 2016) was used to examine the relationship between dependent session variables and independent session variables as well as game characteristics and demographic information. Regression tree algorithms (Lewis, 2000) were used to further explain the relationships between dependent and independent variables. The programming language *Python* was used for statistical analysis (Van Rossum & Drake 1995). The authors chose the number of bets in a session and the session theoretical loss as dependent variables.

## Results

Table 1 reports the game characteristics for each of the six game groups. The numbers were computed based on the raw data collected between November 27, 2020 and April 15, 2021. In total, there were 598 different games and for each game, the respective characteristics were computed. Most games were slots. Blackjack, live blackjack and roulette were standalone games. In total, more than one billion single bets were analyzed (n = 1,108,447,229). This was the sum of all the numbers in the row ‘number of bets’. Out of these, 96.7% bets were made on slots (1,071,817, 666/1,108,447,229). The next most frequent game group was live roulette with 1.7% of all bets (19,054,053/1,108,447,229).

The smallest average bet per single game was made on slots (€1.15) and the largest average bet per single game was made in live blackjack (€49.83). This was also true for the average win for a single game, the standard deviation of the bet, and the standard deviation of the win for a single game. The average win on slots games for a single game was €1.15. The average win for live blackjack for a single game played was €48.31. In relation to hit frequency, the average slots player won every third game. Live blackjack players won on average at every second game. The RTP varied between 96% and 98%. Blackjack and video poker games paid back 98% of the wagers in the form of winnings. The respective value for slots was 96%.

Sessions for slots games lasted an average of 67 min whereas for live blackjack and live roulette it was 36 min. The event frequency between two slot games was an average 6.14 compared to 10 s between two video poker games and 76 s between two games of live blackjack. With 40.87 s and 50.92 s, slots games and video poker had the lowest standard deviations in relation the event frequency. Live blackjack had the largest standard deviation (99.22 s).

**Table 1:** Game characteristics for each game group across all bets between November 27, 2020 and April 15, 2021

*Game characteristic*	*Blackjack*	*Live blackjack*	*Live roulette*	*Roulette*	*Slots games*	*Video poker*
Number of bets	3,188,493	3,868,911	19,054,053	6,058,289	1,071,817 666	4,459,817
Average bet (€)	14.89	49.83	27.83	10.95	1.15	5.10
Average win (€)	14.56	48.31	27.04	10.64	1.11	4.98
Standard deviation win (€)	68.25	406.26	256.73	72.94	34.86	38.43
Standard deviation bet (€)	45.98	266.37	184.11	46.88	6.20	13.74
Hit frequency	2.03	2.01	2.04	2.21	3.43	3.61
Return to player	0.98	0.97	0.97	0.97	0.96	0.98
Average session duration (minutes)	40.32	36.23	36.12	48.05	66.95	48.42
Event frequency (seconds)	15.41	76.55	54.22	20.27	6.14	10.00
Event frequency (standard deviation)	70.38	99.22	89.73	83.91	40.87	50.92

Across the 763,490 sessions in which one game was played, the average session length was 43.58 min, the average loss was €25.69, and the average amount bet was €835.15. The average theoretical loss was €25.90, and the average number of bets was 145.17. The average hit frequency was 3.7 which means that players on average won approximately every fourth game in a session.

Table 2 reports the outcome of a linear regression model with the number of bets in a session as dependent variable and the game characteristics as independent variables. The R^2^ was 7.7%. At the 5% level, each variable contributed significantly to the model. Average bet and standard deviation of the event frequency had negative coefficients. This means that games with larger average bets associated negatively with the number of bets in a session. The larger the variation in the event frequency of a game, the lower the number of bets per session.

**Table 2:** Linear model with number of bets in a session as dependent variable and game characteristics as independent variables

*Game characteristic*	*Coefficient*	*Standard error*	*t*	*p>|t|*	*[0.025*	*0.975]*
Intercept	75.3	25.5	3.0	0.003	25.3	125.3
Average bet (€)	-129.8	9.2	-14.1	0	-147.8	-111.8
Average win (€)	132.9	9.5	14.0	0	114.3	151.5
Standard deviation win (€)	0.1	0.0	2.7	0.007	0.0	0.1
Standard deviation bet (€)	-0.1	0.0	-2.9	0.004	-0.2	0.0
Hit frequency	4.1	0.2	18.9	0	3.7	4.6
Return to player	110.2	26.4	4.2	0	58.5	162.0
Game continuity	3.9	0.0	91.8	0	3.8	3.9
Event frequency	1.6	0.1	12.2	0	1.4	1.9
Standard deviation event frequency	-3.9	0.1	-64.1	0	-4.0	-3.8

In the next step, session-specific metrics and demographic information were added to the list of independent variables. The R^2^ of this model with 18 independent variables was 8.8%. The percentage of the variance explained by the model which also included the session-specific metrics and demographics was therefore significantly larger compared to the model which only included the game characteristics which explained 7.7% of the variance of the dependent variable (F = 1,221, *p* < 0.001).

Table 3 shows the linear model with number of bets in a session as the dependent variable and game characteristics, session-specific metrics, and demographic information as the independent variables. Except for one game characteristic (‘standard deviation win’) all independent variables contributed significantly to the explanation of the number of bets in a session. The maximum bet in a session, the average bet in a session, and the standard deviation of the winnings in a session all had negative coefficients. This means that larger values of these independent variables are associated with a lower number of bets in a session. The demographic variables contributed significantly to the explanation of the number of bets in a session. Age and being female had positive coefficients.

**Table 3:** Linear model with number of bets in a session as dependent variable, game characteristics session-specific metrics, and demographic information as independent variables

*Game characteristic*	*Coefficient*	*Standard error*	*t*	*p>|t|*	*[0.025*	*0.975]*
Intercept	109.3	25.4	4.3	0.00	59.6	159.0
Average bet (€)	-139.5	9.1	-15.3	0.00	-157.4	-121.7
Average win (€)	143.4	9.5	15.2	0.00	124.9	161.9
Standard deviation win (€)	0.0	0.0	1.6	0.10	0.0	0.1
Standard deviation bet (€)	-0.2	0.0	-4.2	0.00	-0.3	-0.1
Hit frequency	3.4	0.3	13.0	0.00	2.9	3.9
Return to player	74.5	26.3	2.8	0.01	23.0	126.0
Game continuity	3.8	0.0	91.0	0.00	3.7	3.9
Event frequency	1.7	0.1	13.0	0.00	1.5	2.0
Standard deviation event frequency	-3.9	0.1	-64.3	0.00	-4.0	-3.8
Session hit frequency	0.4	0.1	4.4	0.00	0.2	0.6
Session maximum bet	-0.1	0.0	-18.9	0.00	-0.1	-0.1
Session maximum win	0.1	0.0	91.0	0.00	0.1	0.1
Session average bet	-0.3	0.0	-14.2	0.00	-0.3	-0.2
Session average win	0.8	0.0	38.2	0.00	0.7	0.8
Session standard deviation bet	0.4	0.0	17.2	0.00	0.4	0.5
Session standard deviation win	-0.8	0.0	-61.5	0.00	-0.8	-0.8
Age	0.2	0.0	6.7	0.00	0.2	0.3
Female	18.7	1.2	15.9	0.00	16.4	21.0

In order to understand the importance of the different independent variables, a regression tree was computed. The data were split up into an 80% training and a 20% test set. The R^2^ on the test set was 26%. The first variable chosen by the regression tree was the game characteristic ‘event frequency’. The threshold chosen was 11.79 s. Sessions with a game that had an event frequency lower than 11.79 had on average 241 bets. Sessions with a game that had an event frequency higher than 11.79 had on average 39 bets. The second most important variable was the maximum amount won on a single bet in a session. Sessions with a game that had an event frequency lower than 11.79 and a maximum amount won of more than €38 on average had 505 bets.

A feature importance analysis showed that the overall most important variable was the maximum amount won on a single bet in a session, followed by the event frequency of the game played, followed by the average amount won per game played in a session. All other independent variables’ contribution was negligible in the regression tree model. In order to examine the relationship between game characteristics and the theoretical loss, a linear model with the session theoretical loss as independent variable was computed. The resulting R^2^ was 4.4%.

**Table 4:** Linear model with theoretical loss in a session as dependent variable and game characteristics as independent variables

*Game characteristic*	*Coefficient*	*Standard error*	*t*	*p>|t|*	*[0.025*	*0.975]*
Intercept	75.3	25.5	3.0	0.003	25.3	125.3
Average bet (€)	-129.8	9.2	-14.1	0	-147.8	-111.8
Average win (€)	132.9	9.5	14.0	0	114.3	151.5
Standard deviation win (€)	0.1	0.0	2.7	0.007	0.0	0.1
Standard deviation bet (€)	-0.1	0.0	-2.9	0.004	-0.2	0.0
Hit frequency	4.1	0.2	18.9	0	3.7	4.6
Return to player	110.2	26.4	4.2	0	58.5	162.0
Game continuity	3.9	0.0	91.8	0	3.8	3.9
Event frequency	1.6	0.1	12.2	0	1.4	1.9
Standard deviation event frequency	-3.9	0.1	-64.1	0	-4.0	-3.8

In the next step, session-specific metrics and demographic information were added to the list of independent variables. The R^2^ of this model with 18 independent variables was 34%. The percentage of the variance explained by the latter model which also included the session-specific metrics and demographics was significantly larger compared to the model which only included the game characteristics and explained 4.4% of the variance. The difference between the two models was statistically significant (F = 25,764, *p* < 0.001). The hit frequency of a game, the standard deviation of the event frequency of a game, the session hit frequency, age and being female were not significantly associated with the session theoretical loss.

**Table 5:** Linear model with theoretical loss in a session as dependent variable, game characteristics session-specific metrics, and demographic information as independent variables

*Game characteristic*	*Coefficient*	*Standard error*	*t*	*p>|t|*	*[0.025*	*0.975]*
Intercept	162.5	14.9	10.9	0.00	133.3	191.7
Average bet (€)	72.0	5.4	13.4	0.00	61.5	82.6
Average win (€)	-74.4	5.6	-13.4	0.00	-85.2	-63.5
Standard deviation win (€)	-0.1	0.0	-6.1	0.00	-0.1	-0.1
Standard deviation bet (€)	0.2	0.0	8.5	0.00	0.2	0.3
Hit frequency	-0.2	0.2	-1.4	0.17	-0.5	0.1
Return to player	-163.8	15.4	-10.6	0.00	-194.0	-133.6
Game continuity	0.2	0.0	7.1	0.00	0.1	0.2
Event frequency	-0.5	0.1	-7.0	0.00	-0.7	-0.4
Standard deviation event frequency	0.1	0.0	1.8	0.07	0.0	0.1
Session hit frequency	0.1	0.1	0.9	0.37	-0.1	0.2
Session maximum bet	0.8	0.0	332.9	0.00	0.7	0.8
Session maximum win	0.1	0.0	159.3	0.00	0.1	0.1
Session average bet	2.0	0.0	188.7	0.00	2.0	2.0
Session average win	2.1	0.0	173.1	0.00	2.0	2.1
Session standard deviation bet	-3.4	0.0	-228.2	0.00	-3.4	-3.3
Session standard deviation win	-1.2	0.0	-159.4	0.00	-1.2	-1.2
Age	0.0	0.0	0.0	1.00	0.0	0.0
Female	1.0	0.7	1.5	0.14	-0.3	2.4

## Discussion

The aim of the present study was to explore the relationship between structural game characteristics, demographics, session metrics, and gambling behavior. Gambling behavior was measured utilizing the number of bets and the theoretical loss in a game session. The theoretical loss was chosen based on the simulation study by Auer, Schneeberger and Griffiths (2012). They concluded that theoretical loss is a more reliable measure of gambling intensity than bet size. The number of bets was chosen to be able to compare the results of the present study with those from Leino et al. (2015). Using data provided by a European gambling operator, the present study computed all online gambling sessions in which only one game was played between November 27, 2020 and April 15, 2021. This approach was chosen in order to be able to compute the correlation between each game’s structural characteristics and the gambling intensity. The results of a linear regression analysis showed that 7.7% of the variance in the number of bets placed in a session was explained by the game’s structural characteristics.

Leino et al. (2015) found that 42% of the variation of the number of bets was explained by games structural characteristics. However, their study was based on video lottery terminals (VLTs) in Norway. The respective games also had much lower RTPs, whereas in the present study, none of the game groups had an RTP lower than 96%. In Leino et al.’s (2015) study, the minimum RTP was 84% and the maximum RTP was 93.04%.

The most important structural characteristic with respect to the number of games played in the present study was the event frequency. The higher the event frequency the larger the number of games played in a session. In a systematic review (n = 11 studies), Harris and Griffiths (2018) found that games with faster speeds of play were preferred and rated as more exciting for all gamblers, ranging from non-problem gamblers to problem gamblers. They also found that fast games were particularly appealing to those who reported a gambling problem. Harris and Griffiths (2018) also concluded that behavioral results were more inconsistent across studies, although the general trend supported the notion that games with faster speeds of play encouraged more wagers, longer game play, and caused players (particularly problem gamblers) to experience difficulty in ceasing gambling. The association between speed of play and greater number of wagers was also apparent in the present study.

A game’s average bet was negatively associated with the number of bets in a session. This means that games with typically larger average bets led to sessions with lower number of bets. This is also understandable because players might run out of funds earlier if they play on higher stake games. Other research has also reported that games with larger bet are played for less time (Sharpe et al., 2005).

In the present study, games with larger average wins were associated with a larger number of bets within session. This contradicts Leino et al.’s (2014) findings among VLT players who found that games with less frequent wins and smaller wins were positively associated with more bets made. One reason for that difference could be the data which were used by the two studies. Leino et al. (2014) used data from VLTs whereas the present study used data from online casinos. In both studies, blackjack and roulette were part of the game portfolio. Leino et al. (2014) reported an RTP for blackjack and roulette of 90.34% and 84.80% respectively whereas in the present study the RTP for blackjack and roulette was 98% and 97% respectively. Roulette has a mathematical house-advantage of 2.7% which results in an RTP of 97.3% which is much closer to the numbers reported in the present study. Other research has also concluded that smaller wins are associated with prolonged gambling (e.g., Delfabbro & Winefield, 1999). However, it should be noted that although the study by Delfabbro and Winefield was also based on within-session gambling, the data were collected by visually observing players (rather than using tracking data).

Apart from game characteristics, the present study also associated session metrics such as the average bet, average win, the maximum win, and maximum bet with the number of bets within session. The model quality was significantly improved, although the R^2^ only increased from 7.7 to 8.8%. A subsequent regression tree analysis found that the maximum win within session was the most important metric in explaining of the number of bets in a session.

This means that players who experience a large win at one point of time within session also play more games. To date, gambling research has described chasing losses (Breen and Zuckerman, 1999) as increased gambling after a series of losses. However, chasing winnings has not previously been empirically studied. One reason for larger number of bets in the case of a larger in-session win could simply be that players have more money available and therefore gamble longer. Consequently, games that provide larger wins might lead to longer gambling sessions.

The first node in a regression tree analysis was the structural characteristic of event frequency. Games which on average lasted less than 11.79 s led to gambling sessions with a larger number of bets. Moreover, a ranking of the importance of the independent variables showed that the structural characteristics of event frequency and the maximum win within session were solely responsible for an R^2^ of 26%. The contribution of all other session variables, structural characteristics, and demographic information was minor.

In line with Auer et al. (2012), theoretical loss appears to be a more valid metric of gambling intensity than the number of bets made. The theoretical loss reflects the amount a player would have lost given the amount wagered and the RTP of the respective game. In a linear model, the game characteristics only explained 4.4% of the variation of the theoretical loss within session. A model which also included session metrics as well as demographics explained 34% of the variation of the theoretical loss in a session. Age and gender were not significant. This means that the monetary gambling intensity in a session is poorly explained by a game’s structural characteristics. The maximum amount bet, amount won, the average bet, average win, and standard deviations of bet and win across all rounds played within session are much more predictive of the theoretical loss. Gamblers can control how much they bet at each round, but they cannot control how much they win. However, it appears that the amount won in a session contributes significantly to the monetary gambling intensity.

### Limitations

The present study utilized data from a large online casino operator with players in multiple European countries. The authors believe that this operator’s game portfolio reflects that of a typical online gambling operator. However, the data in the present study were limited to a single operator and it cannot be ruled out that data from other operators would lead to different results. Also, this operator did not offer draw-based games or instant games that are typically offered by online lotteries. Future research should aim to validate the results from the present study with data from different operators and a wider range of countries. It might also be interesting to combine secondary data with self-report data from the players themselves. This would provide important insights into players’ perceptions of structural characteristics and the potential impact on problem gambling. Other factors that could be investigated to see what is associated with session length and theoretical loss in future studies could include such factors as in-session monetary ‘reloading’ of wallets, side bets, and in vivo vs. deferred side bets.

## Conclusions

There is relatively little research concerning the impact of structural characteristics on gambling behavior and problem gambling. This is surprising given that the game is the product which is at the center of the activity. Most research regarding structural characteristics has been based on self-report data or expert opinions. The present study is only the second study to investigate the impact of structural characteristics based on real-world data from actual gamblers and is the first to utilize online casino data. Given the increasing number of countries in Europe that have introduced and legalized online gambling, insights into the influence of structural characteristics on gambling behavior is an important topic. The latest online gambling regulation in the Netherlands requires license applicants to submit a risk assessment of the games they plan to offer. The present study found that only 7.7% of the variance of the number of games played in an online gambling session were explained by the game’s structural characteristics. This means that 92.3% of the variance is explained by other variables (e.g., a gambler’s individual characteristics such as gender, age, etc.). The authors also computed metrics which reflect a players’ session specific experience. A machine learning tree-based algorithm with structural characteristics and session metrics explained 26% of the variance of the number of games played. Further analysis showed that the structural characteristics of event frequency and the maximum win within session were solely responsible for the model quality. This means that potentially excessive gambling could be a consequence of structural characteristics as well as random events (in the present study, a large win). Given this finding, online gambling operators could potentially display pop-up messages after large wins in order to prevent players from subsequent excessive gambling. The pop-up messages could nudge players to take a break or cash out and take their winnings given that personalized feedback to online gamblers based on their actual gambling behavior can help enable behavioral change (Auer & Griffiths, 2014).
